# Sustainable development of national green innovation capacity: a qualitative comparative analysis based on dynamic public data

**DOI:** 10.3389/fpubh.2025.1621654

**Published:** 2025-06-09

**Authors:** Yi Ran, Liu Qijia

**Affiliations:** ^1^Department of Global Trade and Management, ShinHan University, Gyeonggi-do, Republic of Korea; ^2^Mental Health Development Department, Korea-China Economic Development Institute, Gyeonggi University, Seoul, Republic of Korea

**Keywords:** qualitative comparative analysis, green innovation index, clean energy supply, national coverage, policy orientation

## Abstract

**Introduction:**

In the context of intensified global climate change and increasing environmental constraints, green innovation has emerged as a critical pathway for promoting sustainable national development. As leading economies in Asia, China, Japan, and South Korea have demonstrated distinct approaches to green innovation, making them valuable cases for comparative study. This study aims to identify key factors and configuration paths influencing the sustainable development of national green innovation.

**Methods:**

Using the Qualitative Comparative Analysis (QCA) method and national-level public data, this research constructs multi-factor configurational models to examine how government policy, R\&D investment, clean energy supply and demand, renewable energy share, and environmental greening collectively impact green innovation performance. Robustness checks and country-specific coverage analyses were conducted to ensure the reliability of the findings.

**Results:**

The results reveal three dominant configurations driving green innovation: (1) the synergy between government leadership and R\&D investment; (2) dual-driven paths combining clean energy supply and demand; and (3) a synchronized increase in renewable energy share and environmental greening. Country-specific analysis shows that Japan aligns most closely with the first configuration, reflecting its emphasis on industrial structure and policy-driven innovation. China exhibits strong compatibility with the second configuration, highlighting its diversified strategy and market-based mechanisms. South Korea demonstrates the highest coverage in the third configuration, emphasizing clean energy supply and urban greening efforts.

**Discussion:**

This study concludes that sustainable green innovation development depends on the synergy of multiple factors, and that countries should tailor their strategies based on national resource endowments and policy orientations. The research contributes to the literature by extending the configurational approach to green innovation and offers practical insights for designing differentiated and effective green development policies.

## 1 Introduction

**Figure 1:**
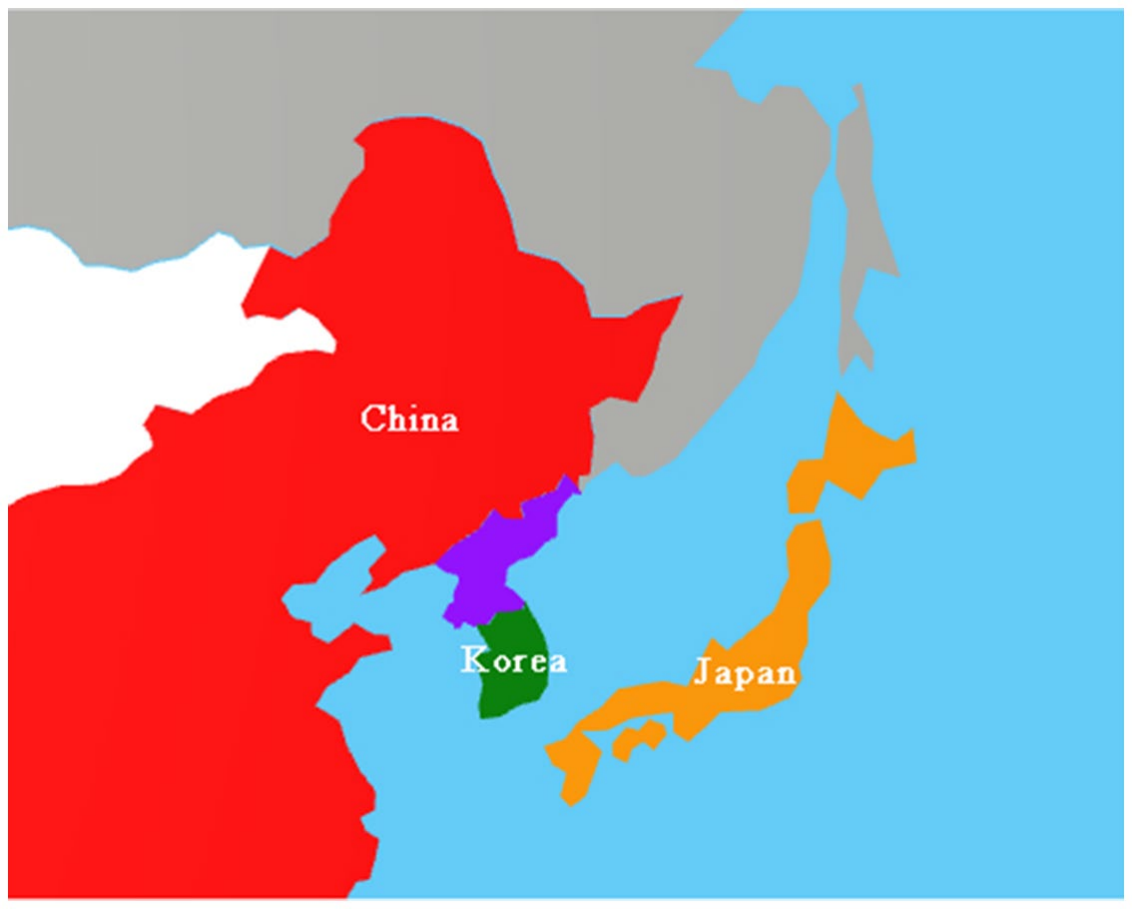
Distribution of different countries.

Against the backdrop of increasingly severe global climate change and resource and environmental constraints, green innovation development has become an important way to promote sustainable economic and social development (1). As important economies in Asia and even globally, China, Japan, and South Korea have attracted much attention for their progress and effectiveness in green innovation development. In recent years, China has actively responded to the call for global green development by formulating a series of green development strategies and policies, promoting green technology innovation and industrial upgrading (2). As shown in Figure 1 as a leader in environmental technology, Japan has accumulated rich experience and technological advantages in areas such as energy conservation, emission reduction, and resource recycling. South Korea is implementing a green growth strategy, vigorously developing renewable energy and green industries, and striving to achieve a win-win situation for both the economy and the environment (3). Green innovation is an important driving force for promoting sustainable economic and social development (4). Through green technology innovation and industrial upgrading, resource conservation and efficient utilization can be achieved, reducing environmental pollution and ecological damage (5). Secondly, green innovation helps to enhance a country’s international competitiveness. Under the trend of global green development, possessing advanced green technologies and industrial systems will become an important component of national competitiveness (6).

At present, research on the influencing factors of green innovation development in China, Japan, and South Korea has made certain progress (7). Current research mainly focuses on government policies, technological innovation, market demand, environmental regulations, and other aspects. However, these studies often focus on the analysis of a single factor or a few factors, ignoring the interactions and linkage effects between multiple factors (8). In addition, although some studies have explored both the supply and demand sides, they are often not comprehensive and in-depth enough (9). This study found that existing research has significant limitations in exploring the influencing factors of green innovation development in China, Japan, and South Korea (10). Specifically, there are relatively few studies that consider the interactions and linkage effects between multiple factors simultaneously (11). Meanwhile, although some studies have explored both the supply and demand sides, they often only focus on one side or a few factors, without comprehensively and deeply exploring the impact of both sides on the development of green innovation. Therefore, in order to comprehensively reveal the internal mechanisms and key paths of green innovation development in China, Japan, and South Korea, this study conducts research from multiple perspectives (12). Based on the above analysis, this study explores from the perspectives of supply and demand (13). Supply factors include government investment, research and development investment, clean energy supply, and other factors. The demand factors include variables such as the proportion of environmental greening rate, the proportion of renewable energy, the proportion of green industries, and the proportion of green output value, which have an impact on the sustainable development of national green innovation (14). These perspectives cover the main aspects of green innovation development and can more comprehensively reveal the internal mechanisms and key paths of green innovation development in China, Japan, and South Korea (15).

In terms of research methods, previous studies have mainly focused on qualitative description, quantitative analysis, and case studies (16). These methods have to some extent revealed the influencing factors and mechanisms of green innovation development in China, Japan, and South Korea, but often overlook the analysis of dynamic changes and configuration effects. Although qualitative description can intuitively reflect phenomena and problems (17). It lacks objectivity and accuracy; Although quantitative analysis can quantify the degree of influence of influencing factors, it is often difficult to reveal the interactions and linkage effects between multiple factors; Although case studies can deeply analyze the characteristics and experiences of specific cases, they are often difficult to promote and generalize (18).

Therefore, this study adopts the dynamic qualitative comparative analysis (QCA) method, combined with spatiotemporal dimensions, to deeply explore the changes in green innovation development in China, Japan, and South Korea in the past decade (19). The dynamic QCA method can reveal the complex relationships and linkage effects between multiple factors, which helps to discover the key paths and combination patterns of green innovation development (20). By constructing a “supply demand” analysis framework and using the dynamic QCA method, this study will explore the synergistic effects of supply and demand factors on regional green innovation on the time axis, and combine one-way ANOVA to examine the distribution differences of configuration country coverage from a spatial dimension. This method can more comprehensively reveal the development status of green innovation in China, Japan, and South Korea, providing useful references and inspirations for subsequent policy formulation and practical operations (21).

From the perspective of supply and demand theory, this study conducted an in-depth exploration of the influencing factors of green innovation development in China, Japan, and South Korea using QCA method. Research has found that factors such as government investment, research and development investment, clean energy supply, proportion of environmental greening rate, proportion of renewable energy, proportion of green industries, and proportion of green output value have mutually influenced and promoted the green innovation development of China, Japan, and South Korea (22). The significance of this study lies in: firstly, filling the limitations of existing research in exploring the influencing factors of green innovation development in China, Japan, and South Korea, and providing new perspectives and methods for research in related fields; Secondly, it reveals the internal mechanism and key path of green innovation development in China, Japan, and South Korea, providing useful references and inspirations for subsequent policy formulation and practical operations; Finally, it has promoted cooperation and exchanges in green innovation development among China, Japan, and South Korea, making positive contributions to jointly addressing climate change and environmental challenges, and achieving sustainable development goals (23). The contribution of this article mainly lies in the following aspects: firstly, from the perspective of supply and demand, it comprehensively considers the interaction and linkage effects between multiple factors; The second is to use dynamic QCA method for data analysis and processing, revealing the internal mechanism and key path of green innovation development in China, Japan, and South Korea; Thirdly, we conducted in-depth discussions and exchanges on the internal mechanisms and key paths of green innovation development in China, Japan, and South Korea, using methods such as spatiotemporal dimensions and one-way ANOVA.

## 2 Green innovation and sustainable development: theory and practice

### 2.1 Green innovation and sustainable development theory

The theory of green innovation and sustainable development is rooted in the classical framework of supply and demand analysis, aiming to reveal how the tension between economic growth and ecological protection can be reconciled through technological innovation and institutional design against the backdrop of increasing resource constraints and environmental pressures. First, from the supply-side perspective, green innovation is seen as an active choice for enterprises to cope with environmental regulation and market competition. Early supply–demand models (e.g., Marshall’s partial equilibrium analysis) pointed out that producers would make production decisions based on factor prices and output prices under the objectives of cost minimization and revenue maximization; after the introduction of environmental protection costs or carbon pricing mechanisms, the relative marginal cost advantage of green technologies became the core driving force for enterprises to adjust the allocation of production factors (24). Schumpeter’s emphasis on the process of ‘creative destruction’ of technological innovation further illustrates that green technology is not only a result of passive adaptation to environmental constraints, but also an endogenous driving force for upgrading industrial structure and forming new growth points (25). In recent years, a large number of empirical studies have shown that by increasing investment in R&D and promotion of clean energy, circular economy and low-carbon processes, the environmental load per unit of output can be effectively reduced, achieving a win-win situation for both the economy and the ecology (26). It can be seen that from the supply side, policy incentives (e.g., green credit, carbon subsidies) and market competition jointly drive the depth and breadth of enterprises’ green innovation upgrading.

On the demand side, consumer preference and public procurement also constitute key pull factors for the diffusion of green innovation (27). In traditional supply and demand curve analyses, a rightward shift in the demand curve implies an increase in market preference for green products, thus forcing producers to seek breakthroughs in technology and processes (28). The ‘responsible consumption and production’ (SDG 12) advocated by the United Nations Sustainable Development Goals (SDGs) provides a macro-orientation for policymakers, encouraging mechanisms such as eco-labelling, green certification and environmental information disclosure to guide consumers from economic rationality to ecological rationality (29). Relevant studies have shown that for every 10 percentage point increase in consumer attention to carbon footprint transparency, the market penetration of green products can be increased by about 5 per cent (30). In addition, the green procurement demand of governments and large organizations has a significant leverage effect: for example, the green public procurement targets set by the EU and Chinese governments, respectively, have accelerated the industrialization of low-carbon building materials and environmentally friendly equipment (31, 32). Therefore, from the demand side, policy guidance and market education work together to expand the market space for green innovations as well as accelerate the process of technological maturity and large-scale application (33).

By organically combining the supply side and the demand side, a two-wheel-driven green innovation sustainable development model can be constructed (34). In this model, the policy design should not only provide R&D subsidies, tax incentives and intellectual property protection on the supply side to reduce the endogenous cost of green innovation, but also expand the effective demand for green products on the demand side by perfecting environmental regulation, strengthening market incentives and enhancing public awareness of environmental protection (35). The ‘Porter’s hypothesis’, put forward by scholars, emphasises that appropriate environmental regulation not only does not inhibit economic growth, but also stimulates the potential for technological innovation, thus achieving a ‘win-win’ situation (36). In practice, China’s ‘Environmental Regulation’ is an example (37). In practice, China’s ‘Peak Carbon - Carbon Neutral’ strategy fully demonstrates the effectiveness of this theoretical framework: Eastern China has achieved a dynamic balance between renewable energy installation and consumption through renewable energy feed-in guarantees on the supply side and smart grid management on the demand side (38). Western China, on the other hand, has promoted the green upgrading of upstream and downstream industrial chains through the ‘dual carbon’ target. In China, the ‘dual-carbon’ target promotes the green upgrading of upstream and downstream industrial chains, and expands the input of market-based capital through green finance (39). The green innovation and sustainable development framework based on the theory of supply and demand not only has a solid theoretical foundation, but also presents good practical effects globally, providing an important reference for promoting high-quality economic development and ecological civilisation in the future (40, 41).

### 2.2 Applied practice of green technology innovation from supply and demand side perspectives

In the context of a dynamically coupled global energy transition and climate governance, the supply-side drivers of green technology innovation manifest principally through government direction and the development of clean-energy infrastructure (42). Firstly, governments employ strategic planning and resource allocation as core instruments—by formulating national green-development blueprints, establishing dedicated innovation funds, and implementing tax incentives and carbon-pricing mechanisms—to provide institutional and financial guarantees for the journey of green technologies from laboratory to demonstration (43). For example, Japan’s Ministry of Economy, Trade and Industry “Basic Hydrogen Strategy” has earmarked ¥350 billion to enhance the hydrogen supply chain, culminating in Kawasaki Heavy Industries’ world-first liquid-hydrogen carrier voyage, thereby underscoring the pivotal role of policy levers in remedying market failures (44). In China, the cumulative investment of ¥2.3 trillion in ultra-high-voltage transmission projects has created the physical backbone for large-scale, cross-regional transmission and consumption of renewables (45). Under South Korea’s “Green New Deal,” ₩73 trillion has been allocated to renewables and hydrogen industries, financing the construction of the nation’s inaugural 20 hydrogen fueling stations and a 1.5 GW offshore wind demonstration site, significantly invigorating domestic equipment manufacturing and systems-integration innovation (46). Secondly, R\&D investment serves as the “engine” of innovation, unleashing multiplier effects via industry-academia-research collaborations. South Korea’s national “Smart Factory” initiative, delivered through partnerships between government, research institutes and leading corporations, has reduced manufacturing energy intensity by 25% and boosted productivity by 20%, with technologies rapidly scaled to commercial application. Although the EU’s Horizon 2020 programme has been a beacon for Europe, South Korea’s “Innovation-Driven State Strategy” has funded the training of over 100,000 green-technology specialists, laying a robust intellectual-capital foundation. Such R\&D outlays not only accelerate core-technology breakthroughs but also generate demonstration effects along the value chain, enhancing market expectations and investor confidence (47). Finally, the construction of clean-energy supply networks—through smart grids, energy storage and demand-response technologies—lowers barriers to renewables integration and fosters green consumption habits among households and enterprises. China’s distributed photovoltaic systems, operating under a self-consumption plus feed-in-tariff model, now cover 20 million homes. South Korea’s Jeju Island smart-grid pilot integrates 500 MW of wind and solar with large-scale storage, achieving a 30% uplift in renewable utilization, thereby creating a “technology→demonstration→optimization→re-innovation” cycle (48).

**Figure 2:**
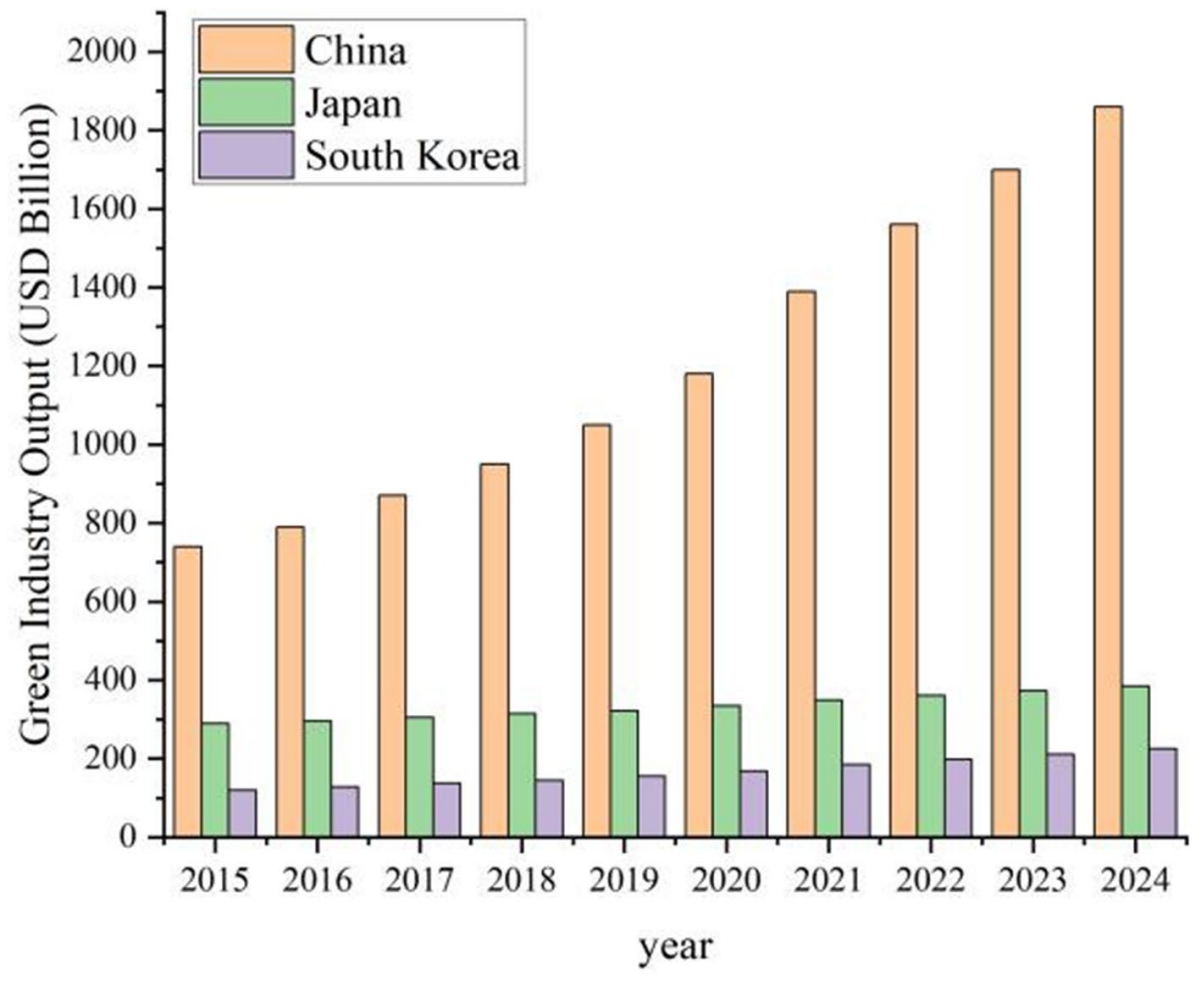
Change in green industrial output.

On the demand side, market pull and societal consensus equally drive green innovation. Firstly, rising environmental greening proportions reflect growing demand for eco-friendly products, prompting governments to intensify green-technology R\&D to satisfy consumer preferences (49). Secondly, an increasing renewables share stimulates research and deployment of renewable-energy technologies, which in turn lowers production costs, enhances energy-use efficiency and optimizes the energy mix (50). As illustrated in Figure 2, green-industry proportions in China, Japan and South Korea are set to continue their ascent through to 2024, indicating an ongoing extension and upgrading of the green value chain—from upstream raw-material recycling, through midstream renewable-equipment manufacturing, to downstream environmental services and market applications. Each segment benefits from policy incentives, technological advances and financial support, thereby solidifying the foundation for green-product R\&D, optimization and diffusion (51). As green products penetrate mainstream markets, corporate innovation vigor and investment appetite visibly strengthen, further promoting cross-sectoral coordination. Transnational collaborations among regional firms and research bodies are also flourishing, facilitating knowledge exchange and resource sharing. Lastly, the rising share of green output reflects the sector’s growing contribution to national economies, unlocking additional policy backing and market opportunities (52). Collectively, these demand-side factors perpetuate continuous green-technology innovation and refinement.

The interplay of supply and demand carries profound implications for sustainable development (53). On one hand, demand-side forces not only directly shape the direction and tempo of green-technology innovation but also exert a market-induced pressure on production structures and corporate decision-making, thereby hastening the transition of technologies from laboratory prototypes to industrial deployment (54). This market-oriented innovation paradigm enhances national competitiveness in the global green economy while boosting value-chain resilience and adaptability. On the other hand, supply-side measures—through policy incentives, fiscal subsidies and infrastructure investments—create a stable environment of technologies and resources that enable consumers and businesses to translate environmental ambitions into concrete purchasing and investment behaviors (55). This “push–pull” synergy accelerates the diffusion and iteration of green technologies and products, engendering a self-reinforcing cycle of sustainable growth. Government and industry must therefore closely monitor evolving consumption patterns, dynamically adjust production and marketing strategies, and reinforce green-industry support policies, refining market access and incentive mechanisms (56). Under dual policy and market stimuli, the deep integration of green-technology innovation and industry development can be achieved, ensuring the harmonious advancement of economic growth and ecological civilization (57).

## 3 Methodology research and theoretical analysis

### 3.1 Qualitative analysis of dynamic QCA

Dynamic qualitative comparative analysis (QCA) is the process of identifying multiple concurrent condition combinations that lead to specific outcomes from complex causal relationships. Unlike traditional statistical methods, dynamic QCA emphasizes the nonlinear relationship between conditions and introduces a time dimension to capture the impact of condition changes across time points on the results (58). This method is particularly suitable for analyzing social and economic phenomena under the influence of multiple factors and dimensions, such as supply side and demand side factors in the transition to a green economy. Its advantage lies in its ability to handle complexity and diversity, revealing the unique contributions of different combinations of conditions to the results, while maintaining in-depth insights into specific cases (57).

In the context of green economy transformation, factors such as government investment, research and development investment, and clean energy supply on the supply side, as well as the proportion of environmental greening rate, renewable energy, green industry, and green output value on the demand side, collectively constitute the core driving force for promoting green development (59). These factors interact with each other and their influence may vary over time. Dynamic QCA analysis can reveal the combination of these conditions at different time points and how they collectively shape the dynamic balance of green supply and demand markets (60). Through in-depth analysis of the impact of these combinations of conditions on green technology innovation, clean energy popularization, and the expansion of green consumption markets, dynamic QCA is helpful in formulating more precise and effective policy measures to promote the sustainable development of the green economy (61).

### 3.2 Analysis and framework construction

**Figure 3:**
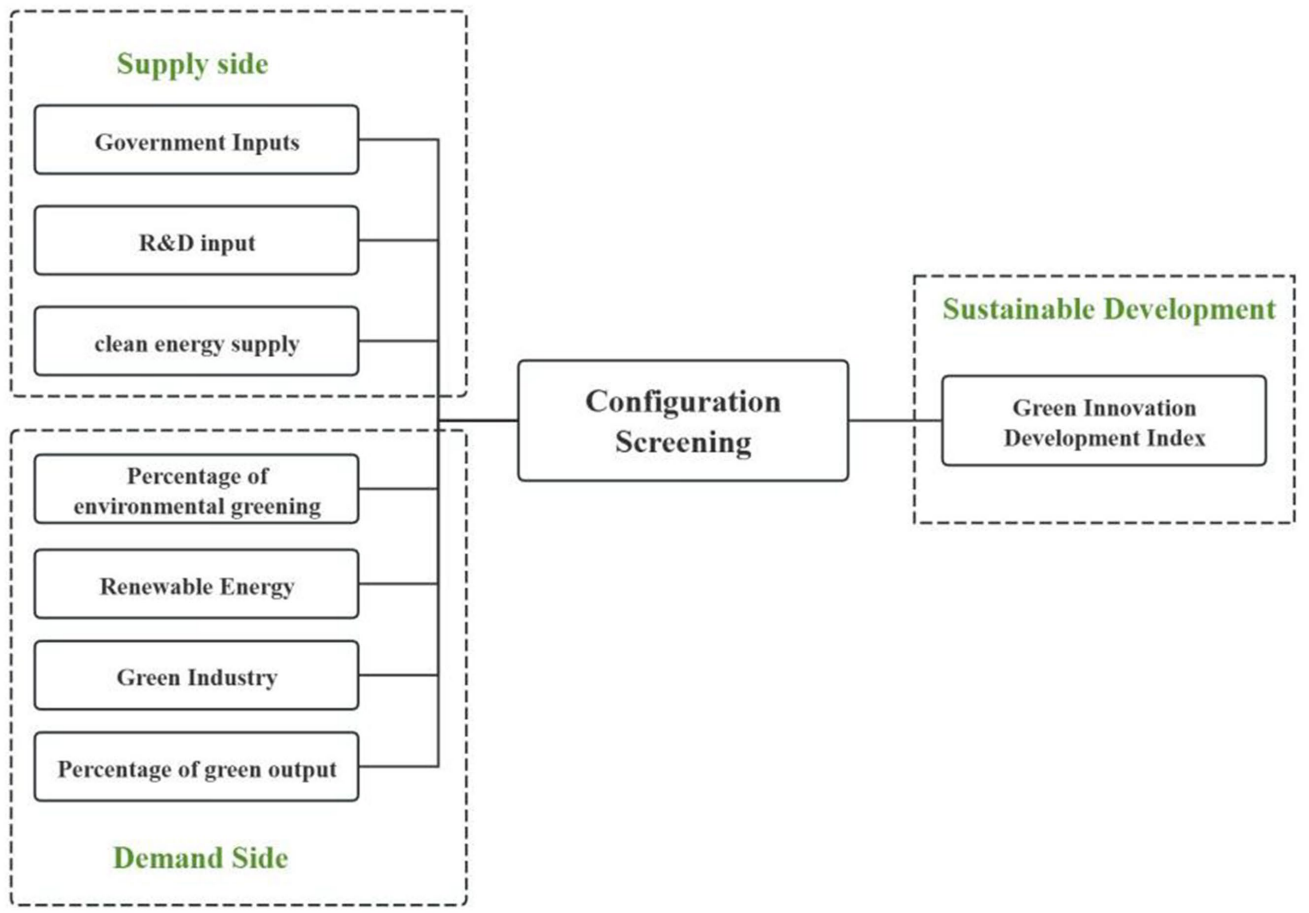
Data framework construction diagram.

Understanding the definitions and sources of various data on the supply and demand sides is crucial when exploring them. As shown in Figure 3. The following is a detailed introduction to government investment, research and development investment, clean energy supply, proportion of environmental greening rate, proportion of renewable energy, proportion of green industries, and proportion of green output value, mainly based on public data from different countries (62).

The supply side data is as follows, government investment: Government investment refers to the government’s financial expenditure in a specific field or project, aimed at promoting the development of that field or solving specific problems.

Data source: Public reports from the national finance department, government budget proposals, and relevant policy documents. For example, China’s government work report will detail the government’s investment in various fields. R&D investment: R&D investment refers to various expenses incurred by enterprises or institutions during the research and development process (63).

Data source: These data are sourced from research funding reports from research institutions and government technology investment statistics. Clean energy supply: Clean energy supply refers to the use of renewable energy sources (such as solar, wind, hydro, etc.) to produce electricity or heat. Data source: These data are usually sourced from energy management departments of various countries. For example, China’s National Energy Administration regularly releases statistical data on renewable energy installed capacity and power generation (64).

The demand side data is as follows, the proportion of environmental greening rate: The proportion of environmental greening rate refers to the percentage of green land area in a region to the total land area. Data source: Statistical data from the urban planning department. Proportion of renewable energy: The proportion of renewable energy refers to the proportion of renewable energy in the total primary energy consumption. Data sources: These data mainly come from energy management departments, international energy organizations, and relevant research institutions. For example, the International Energy Agency (IEA) regularly releases statistical data on the proportion of renewable energy in countries around the world. Proportion of green industries: The proportion of green industries refers to the proportion of green industries in the national economy. Green industry usually refers to industries that focus on environmental protection, energy conservation, and emission reduction in the production process. Data source: These data mainly come from statistical departments, industry associations, and relevant research institutions in various countries. For example, the International Green Industry Federation releases a report on the development of green industries. Proportion of green output value: The proportion of green output value refers to the proportion of green industry output value in the gross domestic product (GDP). Data source: These data mainly come from the statistical departments of various countries. For example, the green GDP accounting system takes into account the costs of resource depletion and environmental degradation to obtain data on green output value. These data are sourced from public information databases in different countries, and are systematically collected and carefully organized through various official channels such as government websites, international authoritative institutions, and professional research institutions (65). These data not only comprehensively reflect the actual situation of various countries in environmental protection, energy, industry, etc., but also provide important basis for evaluating a country’s sustainable development level. At the same time, they are also indispensable reference information for the government to formulate relevant policies, enterprises to adjust their development strategies, and various sectors of society to promote green transformation.

## 4 Data and sample analysis

### 4.1 Data calibration

Based on the established theoretical framework and previous research results, this study implements a uniform and rigorous calibration process for the 2015–2024 public datasets of China, Japan, and South Korea to ensure the reliability of the subsequent intra-group, inter-group, and overall consistency tests and coverage analyses. Considering the continuous value characteristics of the variables, the direct calibration method is adopted, and the three anchor points of full affiliation, intermediate transition, and no affiliation are defined by 95, 50, and 5% quartiles, respectively, in order to provide precise references for data transformation. On the supply side, the study focuses on three core indicators, namely, the intensity of government investment, the proportion of enterprise R&D investment and the supply of clean energy, to measure the development potential of green economy and the strength of policy promotion; on the demand side, the study focuses on four indicators, namely, the environmental greening coverage rate, the proportion of renewable energy consumption, the proportion of green industry and the proportion of green economic output value, to comprehensively assess the response to the green transition and the practice of green transformation from the demand side of the market. The effectiveness of the green transformation is comprehensively assessed as shown in Table 1.

**Table 1:** Variable calibration.

Calibration	Variable name	Fully affiliated	Intersection	Completely unaffiliated
Outcome variables	Green Innovation Development Index	0.435	0.083	0.044
Condition variable	Government Inputs	0.263	0.074	0.042
	R&D Inputs	0.506	0.41	0.222
	Clean energy supply	42,707	50,643	2,763
	Percentage of environmental greening rate	1519.638	662.95	183.960
	Percentage of renewable energy	3.2	2.1	0.795
	Green Industry	220.313	82.58	26.848
	Green Production Value	351.682	134.23	48.404

### 4.2 Analysis of the necessity of individual conditions

According to the interpretation of Boolean algebra in set theory configuration theory, there is a close correlation between the adjustment distance of QCA (qualitative comparative analysis) panel data and its consistency accuracy. The design principle of QCA states that reducing the adjustment distance is usually accompanied by an improvement in consistency accuracy. The root cause of this phenomenon is that smaller adjustment distances can more finely depict the changes between data points, thereby strengthening the overall consistency characteristics of the data (66). In the study, government investment, research and development investment, and clean energy supply on the supply side should theoretically work together to promote the development of green economy. However, in the comparative research of actual situations, it was found that the consistency level of each year did not reach the threshold of 0.9, indicating that there is no strict necessary relationship between them. This may be due to the combined effects of multiple complex factors, such as policy adjustments, market fluctuations, technological innovations, etc., which result in certain uncertainties and volatility in the actual operation of these variables.

Second, in the demand-side study, this study selects four key indicators, namely, the proportion of environmental greening rate, the proportion of renewable energy, the proportion of green industry and the proportion of green output value, and systematically evaluates their coverage and consistency, respectively. As shown in Table 2, in the sample of Scenario A, the coverage is as high as 0.93, indicating that the data collection is more complete; however, the consistency indicator fails to improve in parallel, always hovering below 0.60, which reflects the weak synergy among the indicators in terms of measurement dimension and time series. In order to further clarify this phenomenon, this study re-analyses the aggregation consistency and total coverage of the 2015–2024 data based on Scenario A and the dimension of Government Inputs in China, Japan and South Korea. As shown in Table 3.

**Table 2:** Analysis of the necessary conditions.

variant	Green Innovation Development Index (Y)				Green Innovation Development Index (~Y).			
	Aggregate consistency	Aggregate coverage	Inter-group consistency	Intra-group consistency	Aggregate Consistency	Aggregate coverage	Inter-group consistency	Intra-group consistency
A	0.67	0.75	0.05	0.08	0.56	0.93	0.01	0.07
~A	0.87	0.66	0.01	0.05	0.61	0.66	0.03	0.03
B	0.73	0.56	0.04	0.06	0.7	0.75	0.03	0.06
~B	0.68	0.62	0.07	0.06	0.6	0.74	0.1	0.07
C	0.78	0.79	0.02	0.07	0.72	0.62	0.02	0.12
~C	0.63	0.45	0.01	0.07	0.85	0.84	0.02	0.05
D	0.75	0.66	0.06	0.06	0.73	0.64	0.1	0.08
~D	0.65	0.49	0.1	0.07	0.71	0.8	0.06	0.06
E	0.7	0.56	0.12	0.03	0.64	0.72	0.14	0.05
~E	0.68	0.56	0.11	0.03	0.68	0.73	0.12	0.04
F	0.82	0.72	0.02	0.05	0.72	0.63	0.07	0.09
~F	0.68	0.47	0.04	0.08	0.71	0.85	0.02	0.05
G	0.76	0.69	0.05	0.06	0.62	0.65	0.08	0.08
~G	0.61	0.49	0.07	0.07	0.75	0.81	0.03	0.05

**Table 3:** Aggregation analysis.

Situation	Causal combination situations	Aggregate	2015	2016	2017	2018	2019	2020	2021	2022	2023	2024
a	A and Y	Aggregate consistency	0.68	0.67	0.61	0.62	0.60	0.54	0.25	0.35	0.47	0.51
		Aggregate coverage	0.87	0.86	0.79	0.85	0.86	0.87	0.98	0.96	0.94	0.93

In the 2021 data sample, the coverage has further increased to 0.98 compared with the previous period, indicating that the completeness of the indicator data has been significantly guaranteed; however, the consistency value has plummeted to 0.25, revealing a serious deviation in the correlation and synergistic evolution among the indicators. Taking into account the macro background and the evolution of national governments’ fiscal policies in that year, this study, through literature review and policy tracking, found that in 2021, countries generally adopted stricter preventive and control measures in response to the second and multiple rebounds of the new crown epidemic, including wide-scale blockades, cross-border controls, and restrictions on social activities, among other things. These measures have forced financial resources to focus on public health, social assistance and epidemic prevention and control, and government funding and policy support for green innovation has generally shrunk, resulting in varying degrees of impact on green technology R&D and market promotion.

On the demand side, end-use markets and consumer demand for green products and services have shrunk sharply as a result of the rising economic uncertainty caused by the epidemic, and companies have become more cautious in their production capacity layout and investment decisions, further exacerbating the divergence between the indicators. Hardware indicators such as the environmental greening rate and renewable energy installed capacity have remained relatively stable or even increased slightly as a result of the medium- and long-term planning that has been initiated. The market-driven green industry and green output ratios have fallen sharply due to a gap in demand, leading to a significant reduction in overall consistency. This disconnect between ‘hardware’ and ‘software’ indicators warns of the fragile connection between supply and demand in the face of short-term shocks.

This study reveals the profound impact of external shocks on the synergistic effects of green development policies by systematically analysing the coverage and consistency indicators under different scenarios and time series. Only by strengthening data completeness and indicator synergies during periods of macroturbulence, and through resilient policy design and multilevel collaboration, can we effectively coordinate the rhythms of supply and demand for green development and ensure the sound achievement of green economy goals.

### 4.3 Configuration analysis

The configuration obtained by qualitative comparative analysis (QCA) method can be used to conduct in-depth analysis of its impact on the green innovation index, thereby revealing the key factors driving the development of green innovation and their interaction mechanisms. According to the data shown in Table 4, Firstly, the first configuration may focus on the synergistic effect of government led and R&D investment. Under this configuration, the government has effectively promoted the research and innovation of green technologies through policy guidance and financial investment. The consistency in the configuration is 0.900, the original coverage is 0.402, and the unique coverage is 0.081, indicating high consistency under different schemes, but limited coverage. At the same time, the R&D investment of enterprises has actively responded to the government’s call, forming a positive interaction between the government and enterprises. This synergistic effect not only accelerates the commercialization process of green technology, but also significantly enhances the green innovation index, promoting the green development of the entire society.

**Table 4:** Combination stability analysis.

Conditional variables	Parameterisation1	Parameterisation2	Parameterisation3
(A) Government inputs			
(B) R&D inputs			⊗
(C) Clean energy supply	●	●	●
(D) Percentage of environmental greening rate		●	⊗
(E) Percentage of renewable energy		●	●
(F) Green industry	●	●	
(G) Green production value	●	●	
Consistency	0.905	0.901	0.903
Original coverage	0.321	0.402	0.202
Unique coverage	0.073	0.112	0.043
PRI	0.702	0.712	0.704
Inter-group consistency adjusted distance	0.024	0.021	0.012
Intra-group consistency-adjusted distance	0.035	0.038	0.031
Overall PRI	0.708		
Overall consistency	0.903		
Overall coverage	0.411		

Secondly, the second configuration may emphasize the dual drive of clean energy supply on the supply side and demand side. The consistency under the dual driving combination of clean energy supply and demand is slightly low at 0.867, with original coverage and unique coverage of 0.439 and 0.117, respectively, indicating a slightly stronger presence in the dataset but slightly lower consistency. The widespread application of clean energy provides a continuous driving force for green innovation, while environmental greening further enhances the carrying capacity of the ecological environment and creates a favorable external environment for green innovation. This dual driving mechanism not only helps to reduce the cost of green innovation, but also increases the social recognition of green innovation, thus having a positive impact on the green innovation index.

Ultimately, the third configuration mode focuses on the synchronous increase of clean energy supply and renewable energy share. The consistency of this configuration is as high as 0.912, but the original coverage is only 0.259, and the unique coverage is 0.043, indicating that although this variable is unique in the dataset, its coverage is relatively low. Under this model, green industries are increasingly emerging as a new driving force for economic development. The continuous increase in the proportion of green output value intuitively demonstrates the positive role of green innovation in promoting economic growth. This synchronous growth strategy not only catalyzes the deep optimization and upgrading of industrial structure, but also significantly enhances the economic efficiency of green innovation, laying a solid foundation for the steady rise of the green innovation index. However, it is worth noting that through in-depth analysis of relevant data, the study found that the increase in environmental green coverage and the investment in scientific and technological research and development funds have not promoted the accelerated development of green innovation as expected. This unexpected situation suggests that there may be some unrecognized factors affecting the process of green innovation in this study. Possible reasons include but are not limited to: the synergistic effect between environmental greening and technological innovation has not been fully utilized, or the current research and development investment direction and technological path still need to be further adjusted and optimized to better serve the actual needs of green innovation. This discovery also provides a new perspective and direction for future research and policy-making.

### 4.4 Robustness analysis

The key to ensuring the accuracy and reliability of configuration analysis stability lies in precisely regulating the judgment criteria. The stability adjustment strictly raises the threshold for consistency level from 0.85 to 0.90, effectively screening out more stable and reliable configuration configurations, greatly reducing the risk of misjudgment caused by insufficient consistency. At the same time, in the construction of the truth table, the frequency threshold was raised from 1 to 1.5, and the PRI threshold was increased from 0.60 to 0.70. These adjustments significantly enhanced the rigor of the analysis, reduced the influence of accidental factors on the results, and laid a solid foundation for the robustness and reliability of the analysis results. Entering the stage of strengthening standard analysis, we have fully considered the differences in resource endowments among countries, abandoned the practice of directional assumptions, and adopted a flexible approach of “existence or absence” for all conditions. This comprehensive consideration of various possibilities strategy greatly enhances the applicability and accuracy of the analysis results. Table 4 presents the results of this overall configuration analysis, which includes a total of three configurations. In the comparison process with Table 5, the results of configurations 1, 2, and 3 are similar to those in Table 4, showing the stability of the impact of the supply side variables government investment, research and development investment, clean energy supply and demand side variables environmental greening rate proportion, renewable energy proportion, green industry proportion, and green output value proportion on the development of green innovation. This further proves the reliability of the analysis results after adjusting the judgment criteria and constructing the truth table parameters.

**Table 5:** Different combinations of configurations.

Conditional variables	Parameterisation1	Parameterisation2	Parameterisation3
(A) Government inputs	●		
(B) R&D Inputs			⊗
(C) Clean energy supply	●	●	●
(D) Percentage of environmental greening rate		●	⊗
(E) Percentage of renewable energy		●	●
(F) Green industry	●	●	
(G) Green production value	●	●	
Consistency	0.900	0.867	0.912
Original coverage	0.402	0.439	0.259
Unique coverage	0.081	0.117	0.043
PRI	0.714	0.677	0.611
Inter-group consistency adjusted distance	0.022	0.027	0.012
Intra-group consistency-adjusted distance	0.031	0.035	0.035
Overall PRI	0.712		
Overall consistency	0.911		
Overall coverage	0.405		

### 4.5 Analysis of coverage in different countries

The Green Innovation Index of China, South Korea, and Japan is a key indicator for measuring the activity level of green innovation activities in their respective countries. According to the data shown in Table 6, there are significant differences in the performance of the three countries in this field. It is worth noting that configuration 3 showed the highest coverage in South Korea, reaching 0.442, while the coverage in China and Japan was only 0.153 and 0.073, respectively. The significant achievements of South Korea in the field of green innovation may be attributed to its emphasis on domestic clean energy supply and the continuous increase in the proportion of public environmental greening. These factors provide a solid foundation for the vigorous development of green innovation activities in South Korea, not only promoting the research and application of clean energy technologies, but also enhancing public awareness and participation in green living. On the other hand, Configuration 2 has the most outstanding coverage performance in China, reaching 0.279, surpassing Japan and South Korea’s 0.224 and 0.135. This to some extent reflects China’s diversified development strategy in the field of green innovation. From the supply side, the supply of clean energy provides a continuous source of power for green innovation; From the demand side, the joint increase in the proportion of environmental greening rate, renewable energy, green industry, and green output value further promotes the comprehensive prosperity of China’s green innovation field. This synergy between supply and demand has injected strong impetus into the sustained growth of China’s green innovation activities. As for configuration 1, Japan’s coverage in this configuration is as high as 0.572, significantly higher than China and South Korea’s 0.125 and 0.152. This indicates that the changes in Japan’s Green Innovation Index are closely related to factors such as clean energy supply, the proportion of green industries on the supply side, and the proportion of green output value. Japan not only pursues the speed of economic growth in its economic development, but also attaches great importance to the long-term goal of sustainable development. The pursuit of this dual goal has given strong impetus and support to green innovation activities in Japan. The widespread application of clean energy, the rapid development of green industries, and the sustained growth of green output value together constitute the beautiful landscape of Japan’s green innovation field.

**Table 6:** Geographical coverage.

Country coverage	Korea	China	Japan
Configuration 1	0.152	0.125	0.572
Configuration 2	0.135	0.279	0.224
Configuration 3	0.442	0.153	0.073

## 5 Discussion and implication

### 5.1 Conclusion

This study, grounded in a multidimensional perspective of supply–demand theory, employs the method of qualitative comparative analysis (QCA) to construct a comprehensive supply–demand analytical framework. It is supplemented by dynamic QCA and one-way analysis of variance, among other statistical techniques, to conduct a systematic examination of green innovation development in China, Japan and South Korea from 2015 to 2024. A unified and rigorous calibration procedure was applied to each nation’s publicly available datasets, ensuring the reliability of consistency and coverage analyses within groups, between groups and overall.

The findings indicate that the synergistic interaction between government leadership and R\&D investment, the dual driving mechanism of clean energy supply and demand, and the concurrent enhancement of clean energy supply and renewable energy share constitute key pathways propelling green innovation in the three countries. These pathways exhibit varying degrees of coverage and influence in each nation, yet all have been essential to the vigorous advancement of green innovation activities. In South Korea, the reinforcement of clean energy supply and the increase in the proportion of green urban coverage have markedly advanced the green innovation index, reflecting South Korea’s notable achievements and sustained endeavour in this domain. In China, the coordinated efforts at both the supply and demand ends—especially the joint increase in clean energy supply, urban green coverage, renewable energy share, green industry share and green output value share—have injected robust impetus into the comprehensive prosperity of China’s green innovation sector. In Japan, the widespread adoption of clean energy, the rapid development of green industries and the continuous growth in green output value have collectively driven substantial progress in green innovation activities, illustrating Japan’s steadfast commitment to sustainable development objectives alongside its pursuit of economic growth.

Theoretically, this study enriches the multidimensional complementary perspective on the mechanisms influencing green innovation; methodologically, it innovatively integrates dynamic QCA with analysis of variance, accommodating both temporal evolution and cross-national comparison; and practically, it delineates three replicable key pathways and national governance characteristics, offering targeted recommendations for policymakers to optimise green innovation support systems under varying national contexts. Future research might further validate the configurational patterns identified herein and assess their applicability at more granular regional or industry levels.

### 5.2 Theoretical implication

In the study of green innovation development in China, Japan and South Korea, this study breaks through the limitations of the previous single perspective by placing the three main actors, namely government, enterprises and market, under the overall framework of the unified ‘supply and demand’ theory, and systematically reveals their synergistic roles in the process of green technology R&D, application and marketization. The role of green technology in the process of green technology R&D, application and marketization is systematically revealed. Unlike previous studies that mostly start from policy effects or market demand, this study fills the gap of existing literature that ignores the interaction between supply and demand and lacks overall linkage analysis, providing a new perspective for an in-depth understanding of the synergistic mechanism of the three main actors.

Based on dynamic qualitative comparative analysis (QCA), this study clarifies for the first time that close synergy between supply and demand is the core path to enhance the green innovation index, and elucidates that the benign interaction of ‘government policy guidance + enterprise R&D investment’ can accelerate the commercialization process of green technologies. Meanwhile, through the comparison of cross-country allocation paths, this study also verifies the driving mechanism of clean energy supply on green innovation.

Finally, to address the shortcomings of traditional qualitative or regression methods that are highly static and difficult to capture the evolutionary process of green innovation, this study innovatively combines dynamic quantitative analysis with one-way ANOVA, which not only realizes the cross-period time series tracking of causal configurations, but also completes the comparison of the spatial coverage of China, Japan and Korea. This methodological innovation not only overcomes the problem of fragmentation of causal mechanisms in previous studies, but also lays a solid methodological foundation for future validation of key configuration patterns and deepening of green innovation evolution mechanisms at a more refined regional or industrial level.

### 5.3 Managerial implication

Given that research has shown that synergy between government guidance and enterprise R&D inputs can significantly enhance green innovation performance, we should further break down sectoral barriers in the future and create a closed loop of integrated policies on finance, science and technology, industry and environmental protection. It is recommended to set up an inter-departmental ‘Green Innovation Coordination Committee’ to coordinate financial subsidies, science and technology projects, industrial support and environmental protection supervision; implement a ‘gradient-type green subsidy mechanism’, allocating funds in phases according to the technological maturity of the enterprise and the marketization process; smooth the channel of fast review of green intellectual property rights and improve green standardization. Intellectual property rights, improve the green standardization system, and provide institutional protection for technologies moving from the laboratory to industrialization; and encourage regional governments to build green technology transfer platforms with international institutions, lower cross-border proliferation barriers, and enhance the international scale and influence of green innovation.

Based on the path of ‘double-driven clean energy supply and demand’, the policy should give more prominence to demand-side management and market-based incentives. Firstly, improve green consumption incentives - promote green product certification and carbon labelling, set up green consumption points and corresponding tax breaks, to enhance the public’s and enterprises’ willingness to buy green; secondly, for the upstream and downstream of the industrial chain, innovate “green power direct supply” and “green procurement.” Secondly, for the upstream and downstream of the industrial chain, innovate ‘green power direct supply’, ‘green procurement’ and ‘carbon-neutral supply chain finance’ modes to stimulate enterprises to continuously invest in green technologies; at the same time, accelerate the construction of smart grids and distributed energy management systems, and lower the threshold of green energy access; finally, jointly formulate a ‘green technology application roadmap’ with industry associations. Finally, we will work with industry associations to formulate a ‘green technology application roadmap’, specifying short-, medium- and long-term promotion targets and key indicators for various technologies, and guiding R&D resources to accurately meet market demand.

Considering the differences in green innovation paths between China, Japan and South Korea, precise policies should be implemented at the regional and national levels. First of all, we should build a framework of ‘fractional governance of green innovation’ and formulate differentiated policy packages by taking into account the resource endowments and industrial characteristics of each region: for example, eastern China focuses on green industry clusters, and central and western China increases investment in clean energy infrastructure; South Korea deeply integrates the application of clean energy with the construction of green cities; and Japan accelerates the internationalisation of green industries and the leadership of core technologies. Japan is accelerating the internationalisation of green industries and leading in core technologies. Secondly, to build a digital ‘green innovation dynamic monitoring and early warning system’, using big data to track the progress of technology, market changes and the effect of policy implementation in real time, and dynamically optimise resource allocation and policy combinations through scenario simulation and performance evaluation, so as to enhance the resilience and adaptability of the green innovation system to external shocks such as epidemics and fluctuations in energy prices. By focusing on both differentiated and dynamic governance, the precision, flexibility and sustainability of green innovation policies can be enhanced, laying a solid foundation for long-term green transformation.

### 5.4 Limitation and future research

In the process of exploring the development of green innovation in China, Japan, and South Korea, although this study adopts the dynamic qualitative comparative analysis (QCA) method and comprehensively considers multiple factors from the perspective of supply and demand theory, there are still some limitations. Firstly, the limitations of data acquisition and processing cannot be ignored. Due to differences in data statistical standards and calibers among different countries, there may be certain deviations in data comparison and analysis. In addition, some key data may be missing due to confidentiality or difficulty in obtaining, thereby affecting the comprehensiveness and accuracy of the research. Secondly, this study is mainly based on existing theories and literature for deduction and analysis, which may have certain subjectivity and limitations. Although we strive to maintain objectivity and impartiality, interpreting data and results may still be influenced by personal experience and knowledge background. In the future, in order to further explore the internal mechanisms and key paths of green innovation development in China, Japan, and South Korea, research will improve from the following aspects: first, strengthen the standardization and normalization of data collection and processing, and improve the comparability and accuracy of data. Secondly, introduce more empirical research and case analysis to verify and supplement the results of theoretical derivation. Finally, expand research perspectives and methods, and combine theories and methods from other related disciplines such as environmental economics and energy policy research to conduct interdisciplinary comprehensive analysis. By continuously improving and expanding research methods, research can more comprehensively reveal the inherent laws and trends of green innovation development in China, Japan, and South Korea, providing stronger support and guidance for policy formulation and practical operations.

## Data Availability

Publicly available datasets were analyzed in this study. This data can be found at: https://www.un.org/zh/library/page/databases.
